# Supplementary material for: Interpretable classification of Alzheimer’s disease pathologies with a convolutional neural network pipeline
Source: Nat Commun. 2019 May 15;10:2173. doi: 10.1038/s41467-019-10212-1 (PMC6520374; doi:10.1038/s41467-019-10212-1)
Supplement: Supplementary file 1 — Supplementary Information [file 41467_2019_10212_MOESM1_ESM.pdf]

Supplementary Information for
Interpretable classification of Alzheimer’s disease pathologies with a convolutional neural network pipeline

Ziqi Tang, et al.

*To whom correspondence should be addressed, keiser@keiserlab.org; bndugger@ucdavis.edu

Supplementary Figures

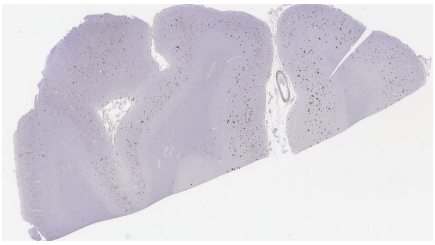

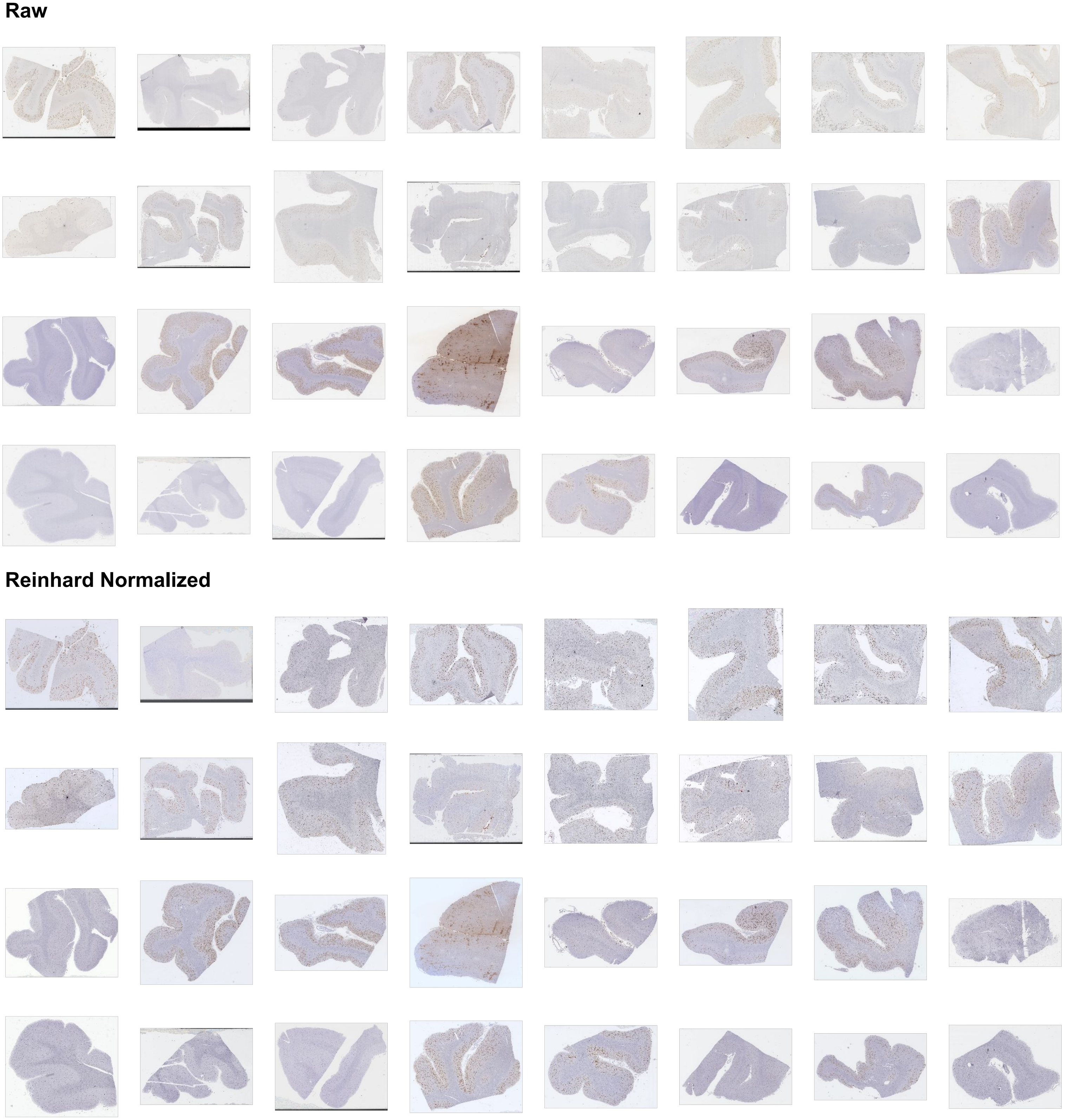

Supplementary Figure 1 Whole slide image color normalization by the method of Reinhard et. al.

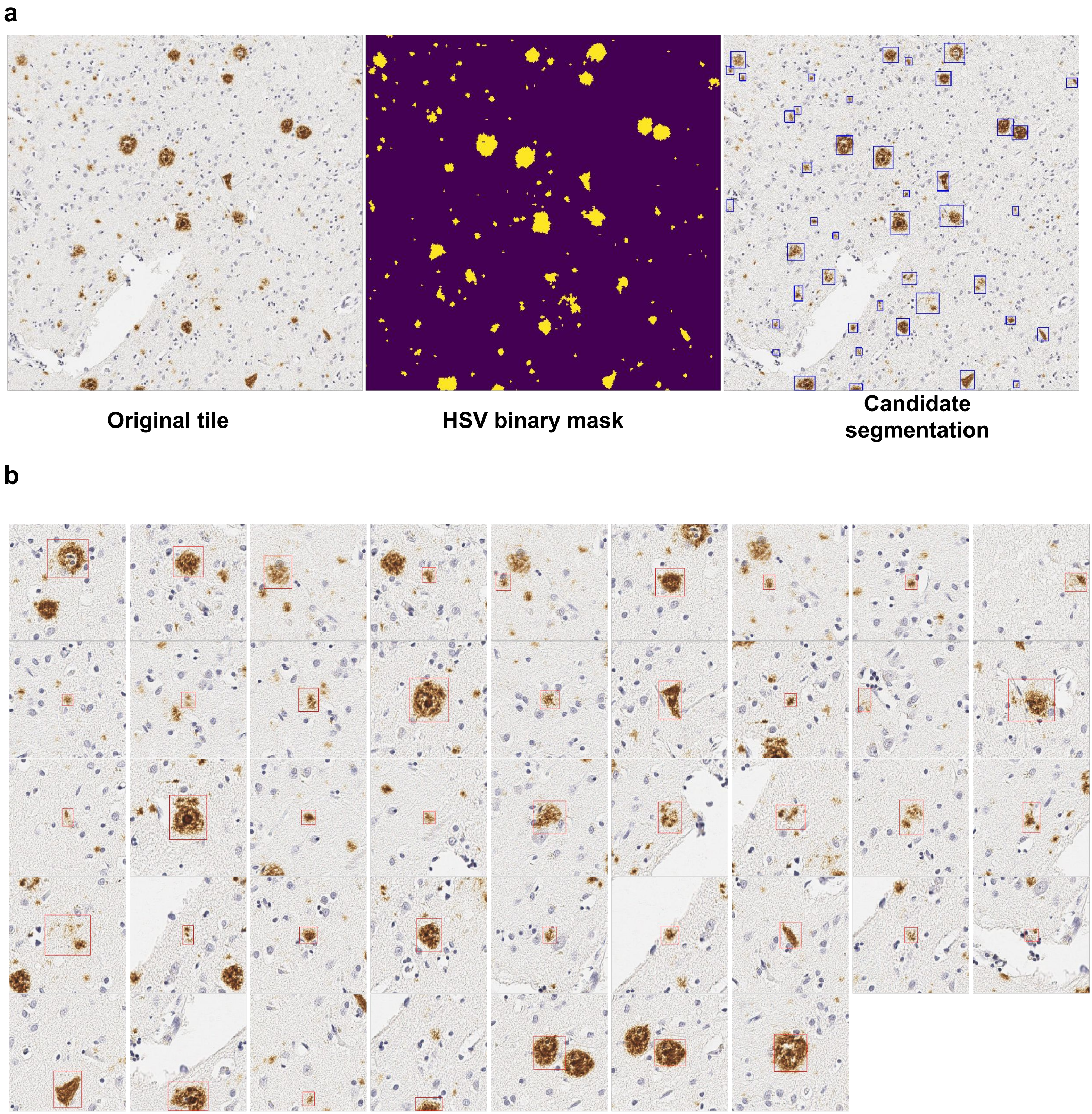

Supplementary Figure 2 Automatic plaque-candidate segmentation. **a.** A permissive HSV-filter is applied to the original tile and smoothed, providing a binary mask for segmentation. Continuous regions are automatically bounded in minimum boxes and subsequently center cropped. **b.** Resulting center-cropped images with candidate plaques.

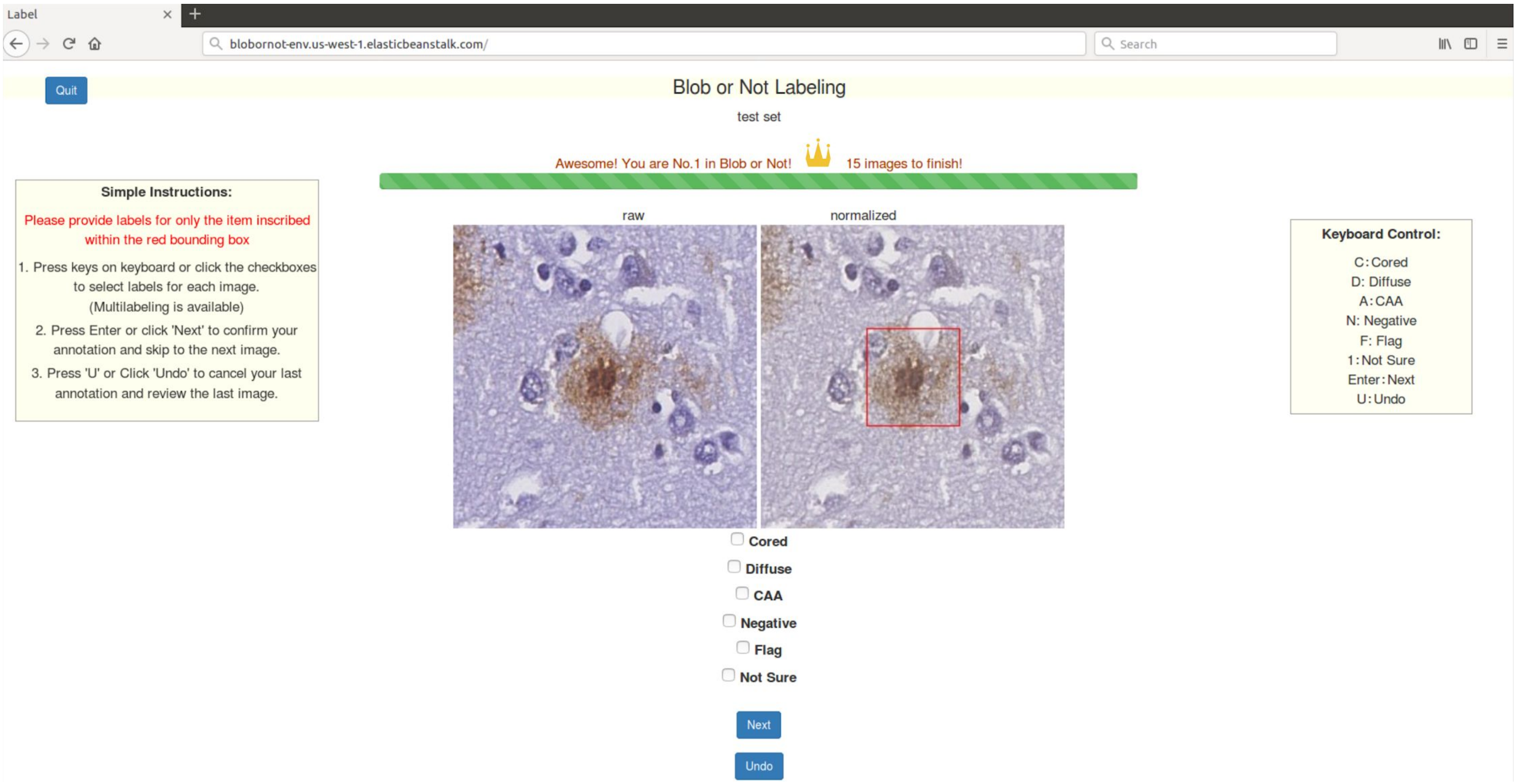

Supplementary Figure 3 Live screenshot of the custom web platform for rapid image annotation used in this study. Images before and after normalization are shown for comparison. A bounding box in the normalized image specifies which specific blob being labeled. Several elements of gamification, such as leveling, achievement badges (crown icon), and progress bar filling (green bar) are incorporated to reward and motivate annotation task progress. Authorized annotators used a rapid keystroke entry format to label candidate plaques. All labels were stored in a SQL database using Amazon Relational Database Service.

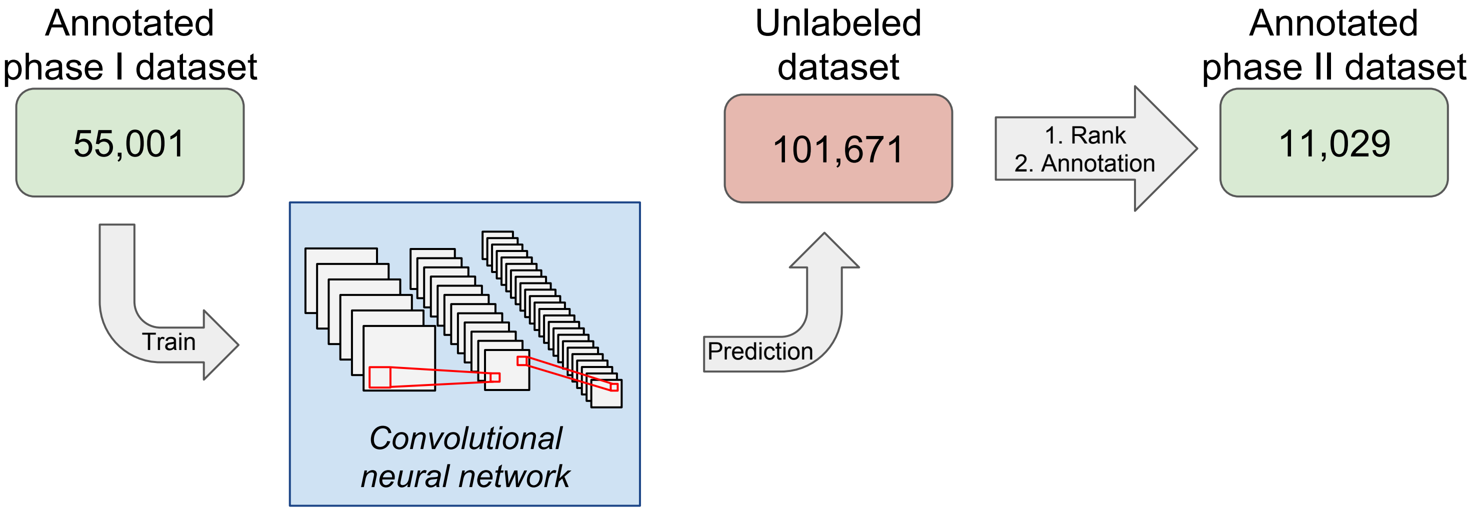

Supplementary Figure 4 Annotation phase II for minor classes enrichment. An intermediate CNN model for plaques classification was trained on the phase I dataset containing 55,001 annotated images. The trained model was applied to another dataset with 101,671 unlabeled images. These unlabeled images were ranked by the prediction confidence for cored plaques or CAAs. 11,029 images with high prediction confidence for cored plaques or CAAs were then labeled by the neuropathologist as annotation phase II.

a

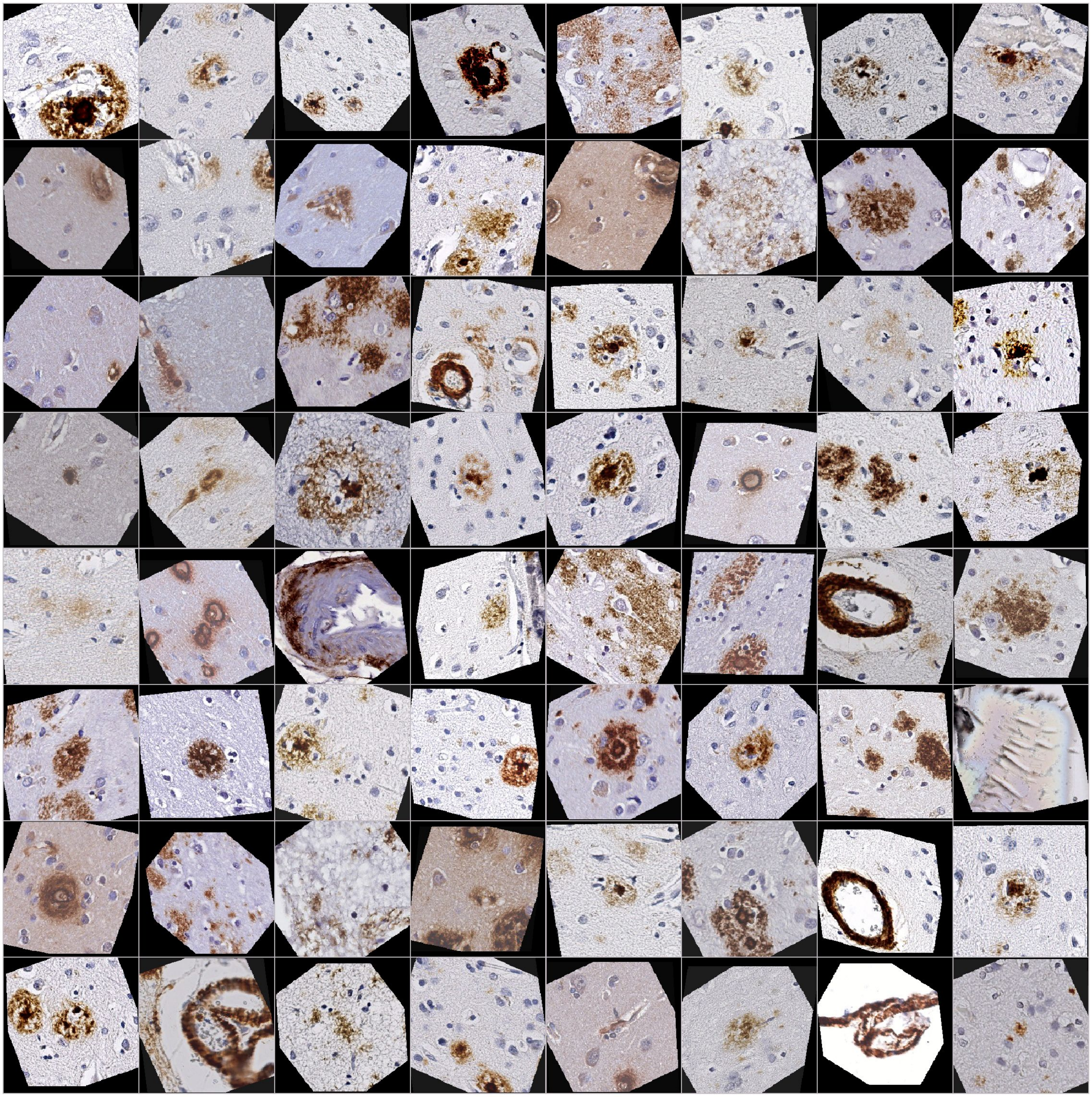

b

Dataset	Cored	Diffuse	CAA	Negative	Total
**Raw**	2,141 (3.49 %)	48,123 (78.41%)	2,227 (3.63%)	3462 (5.64%)	61,370
**Upsampling**	49,411 (31.83 %)	67,008 (43.16 %)	49,170 (31.67 %)	3462 (2.23%)	155,239

Supplementary Figure 5 CNN training was performed with real time augmentation and minority class upsampling. **a.** Training examples shown with data-augmentation, including random flips, rotations, zooms, and color jitter. **b.** Training was evaluated with and without minority-class upsampling.

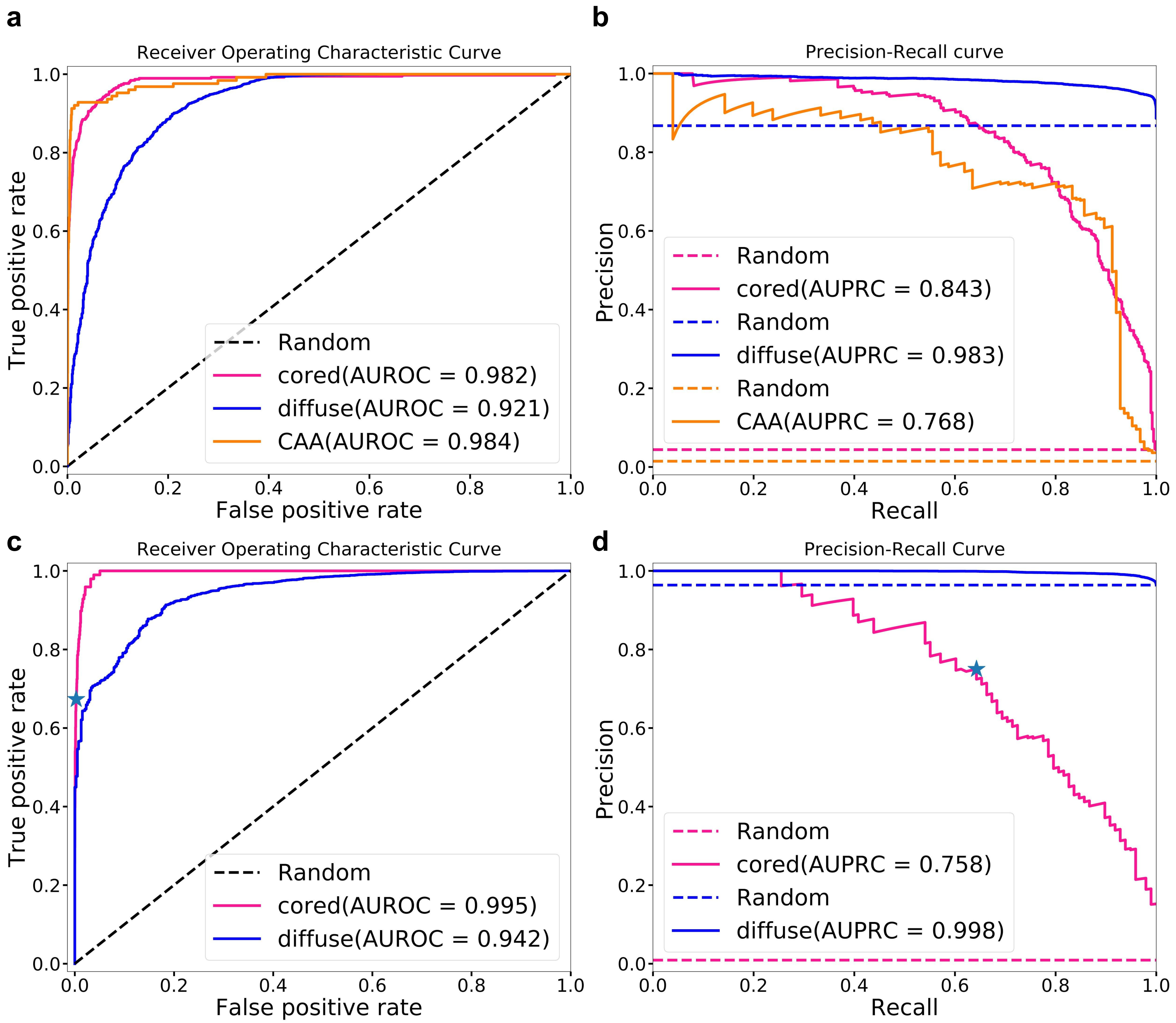

Supplementary Figure 6 Prediction performance without color normalization. **a**. Receiver operator characteristic curve (on validation set). **b.** Precision-recall curve (on validation set). **c**. Receiver operator characteristic curve (on hold-out set). **d.** Precision-recall curve (on hold-out set). Source data are provided as a Source Date file.

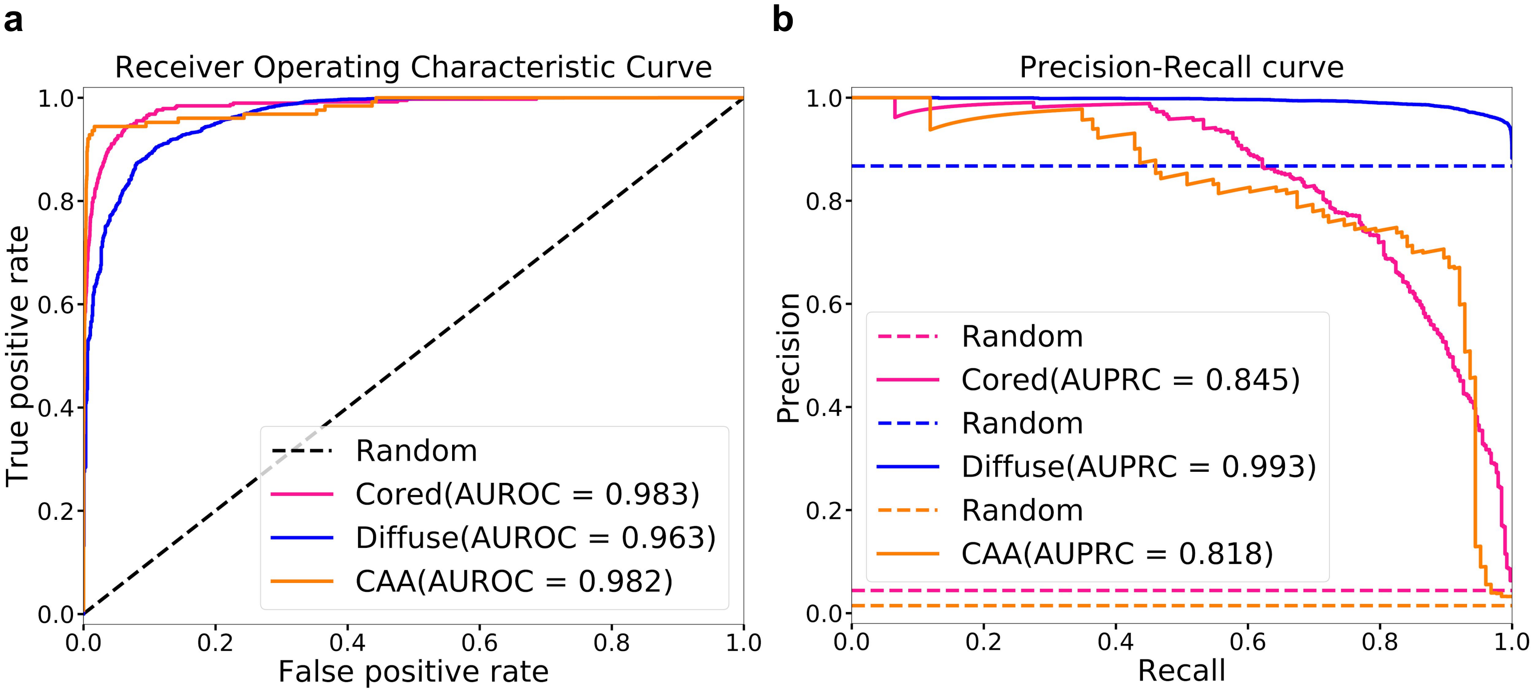

Supplementary Figure 7 Prediction performance on validation set. **a**. Receiver operator characteristic curve. **b.** Precision-recall curve. Source data are provided as a Source Date file.

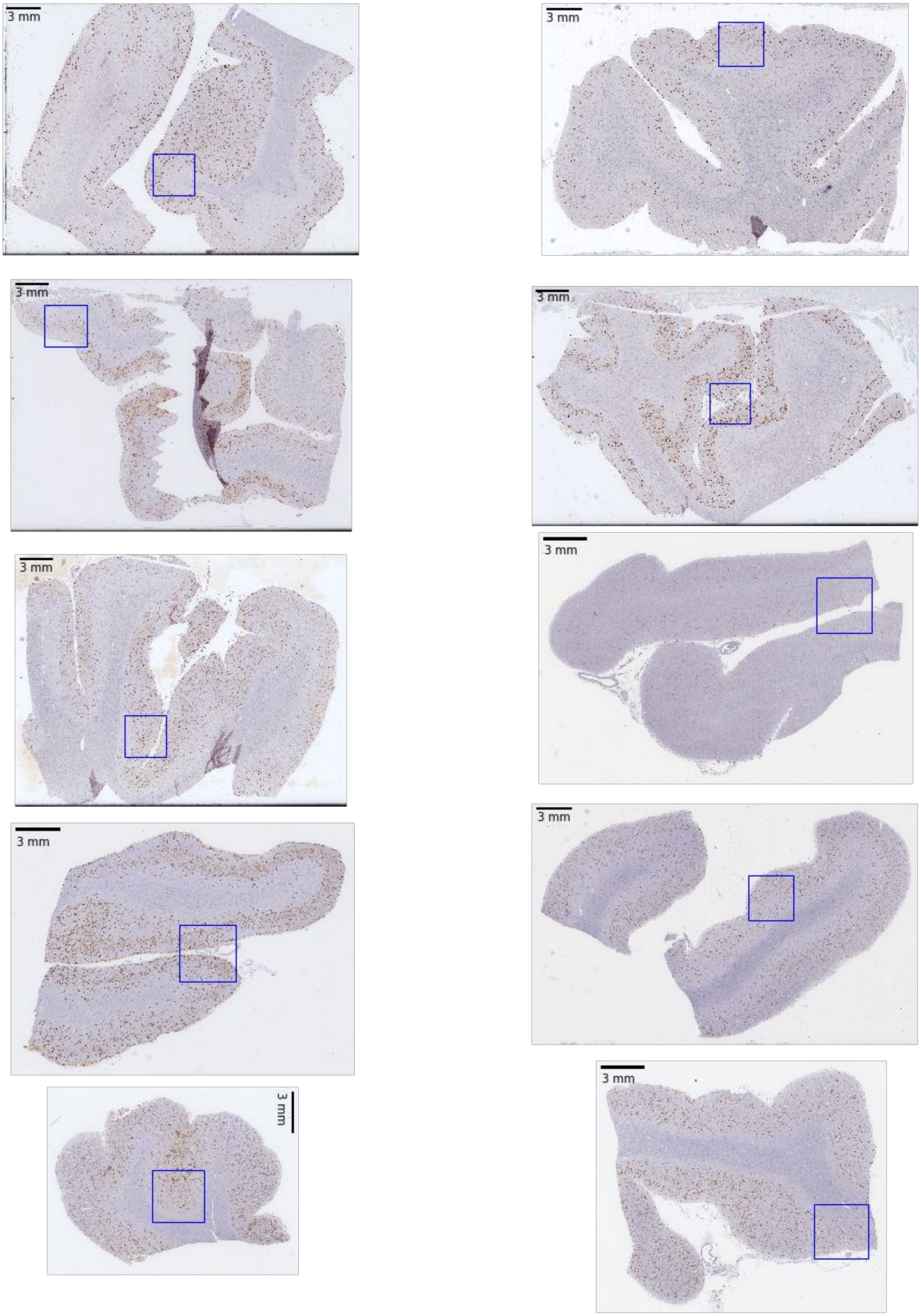

Supplementary Figure 8 Hold-out test set WSIs. Continuous regions of interest (corresponding to 3.8 x 3.8 mm tissue regions) are outlined in blue. Candidate plaques were extracted using the segmentation script for local analysis.

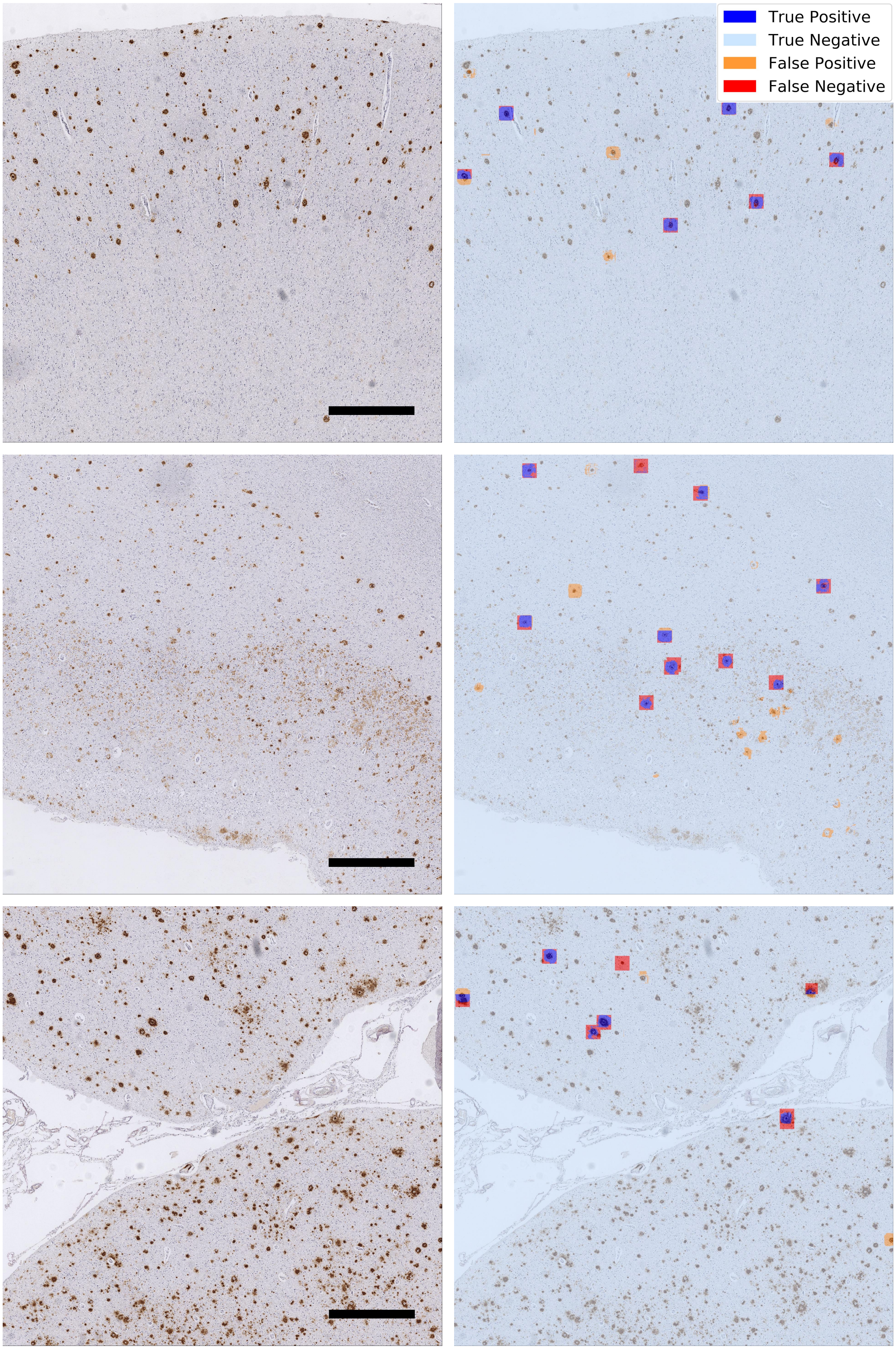

Supplementary Figure 9 Additional examples of visual representation of classification performance on held-out set against ground truth plaque labels. Overlaying prediction confidence maps with expert labeled cored plaque bounding-boxes provides a color-coded method for visualizing network performance. The resulting plot (right column) shows the assessment of agreement between CNN predictions and expert labels, in terms of true positives (blue), false positives (orange), true negatives (cyan), and false negatives (red). Scale bar=750 um.

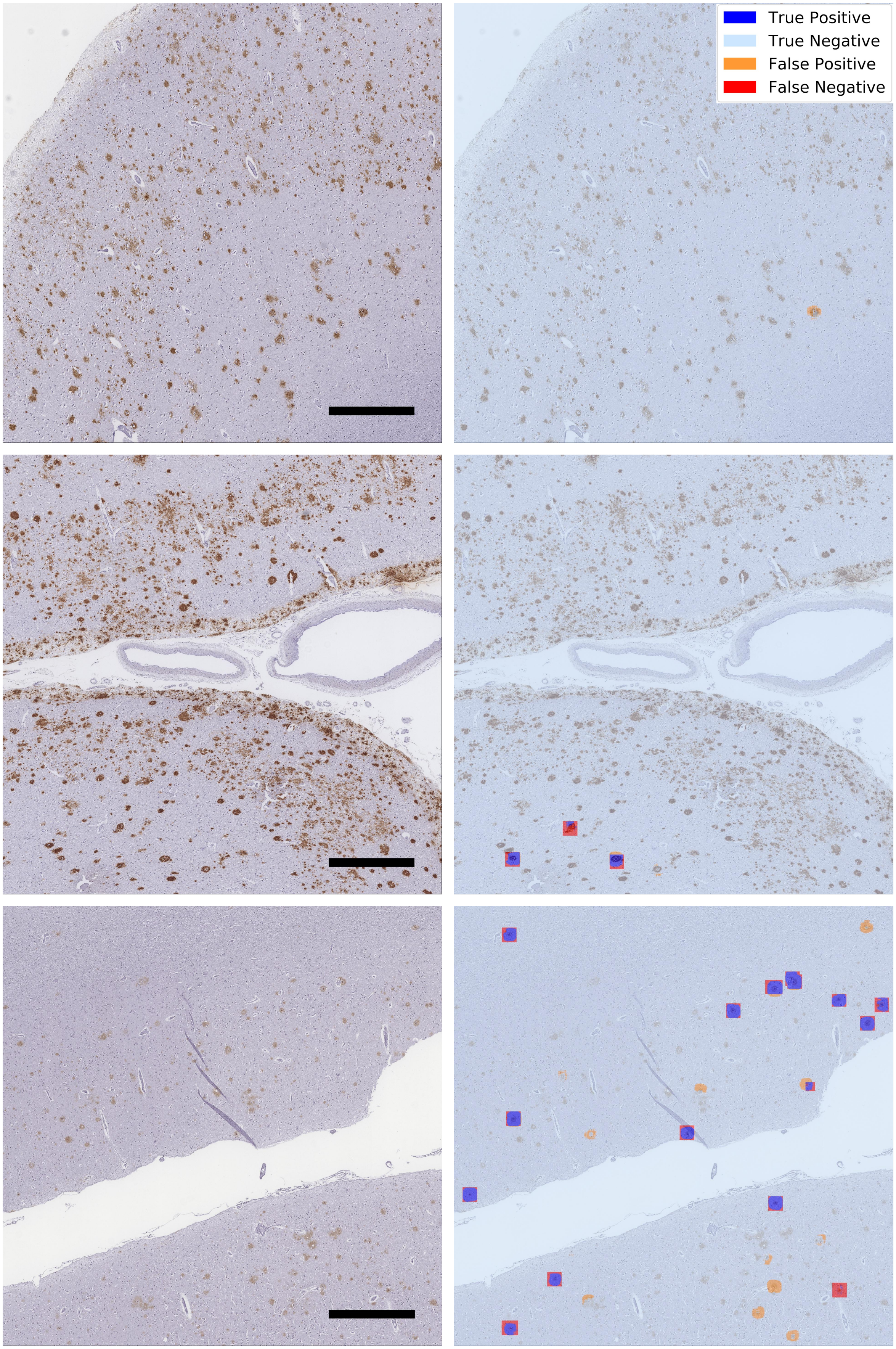

Supplementary Figure 9 continued

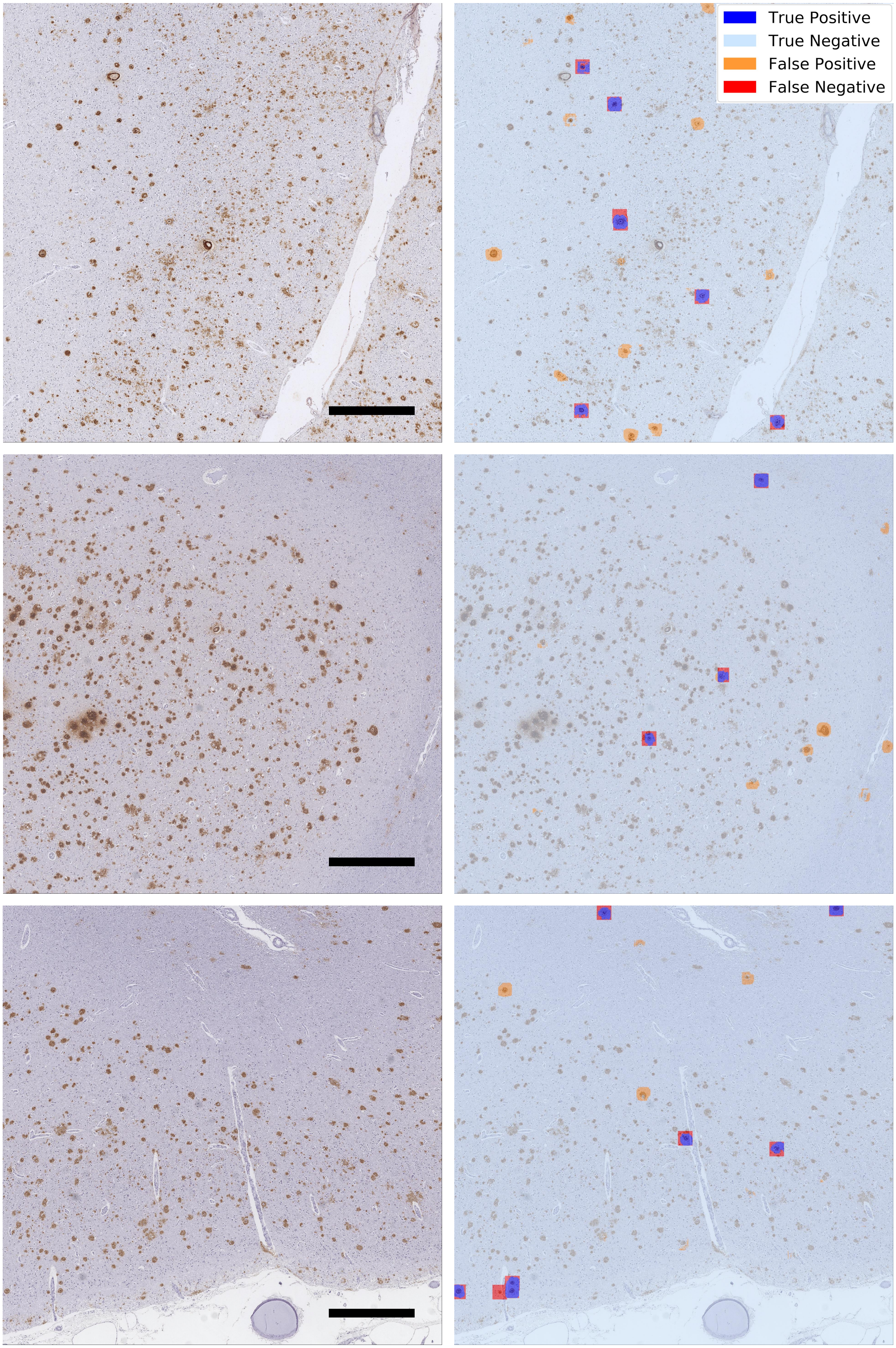

Supplementary Figure 9 continued

a

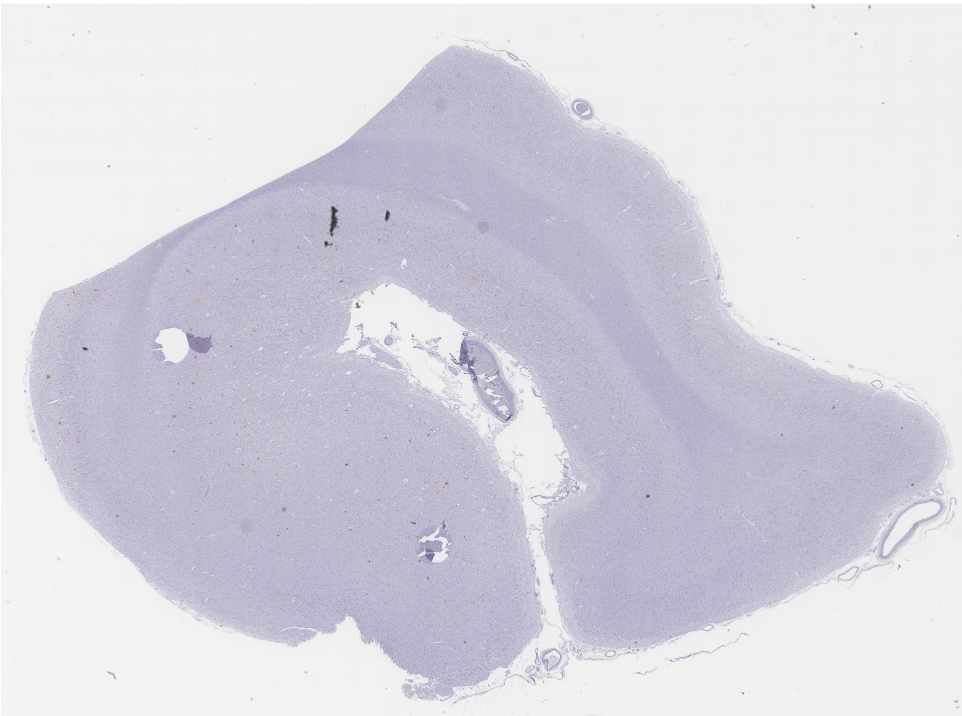

b

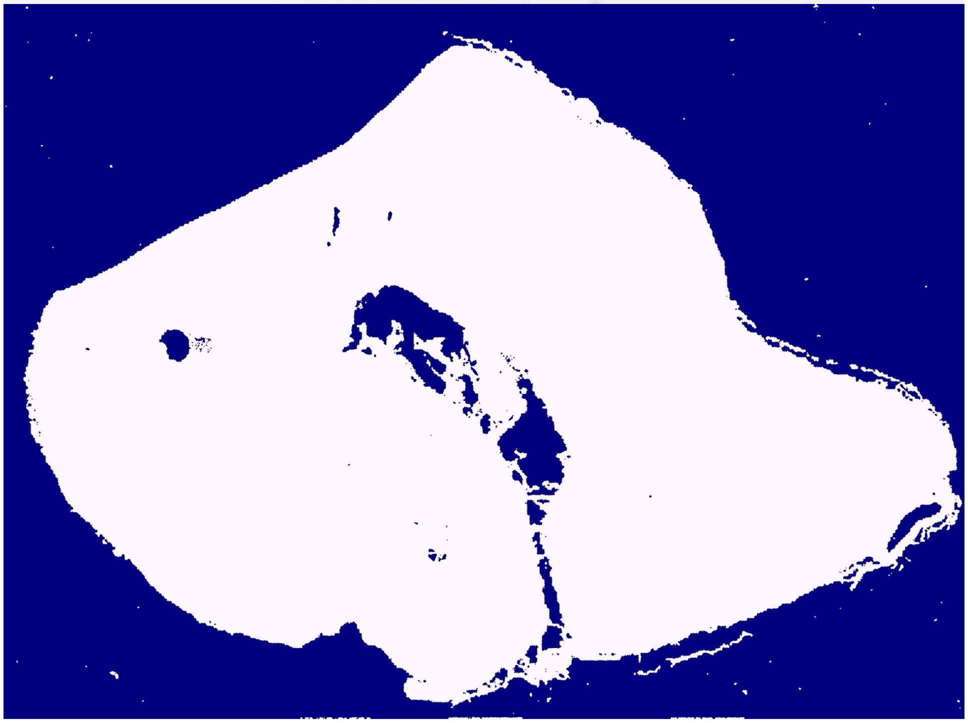

Supplementary Figure 10 Tissue segmentation example. Tissue segmentation was performed in the lightness-chroma-hue (LCH) colorspace utilizing a specific colormask for each WSI. Morphological opening and closing operations were performed to smooth the colormasks, and the tissue areas were calculated as the pixel sum of the refined mask. **a.** IHC stained whole slide image. **b.** Segmented mask.

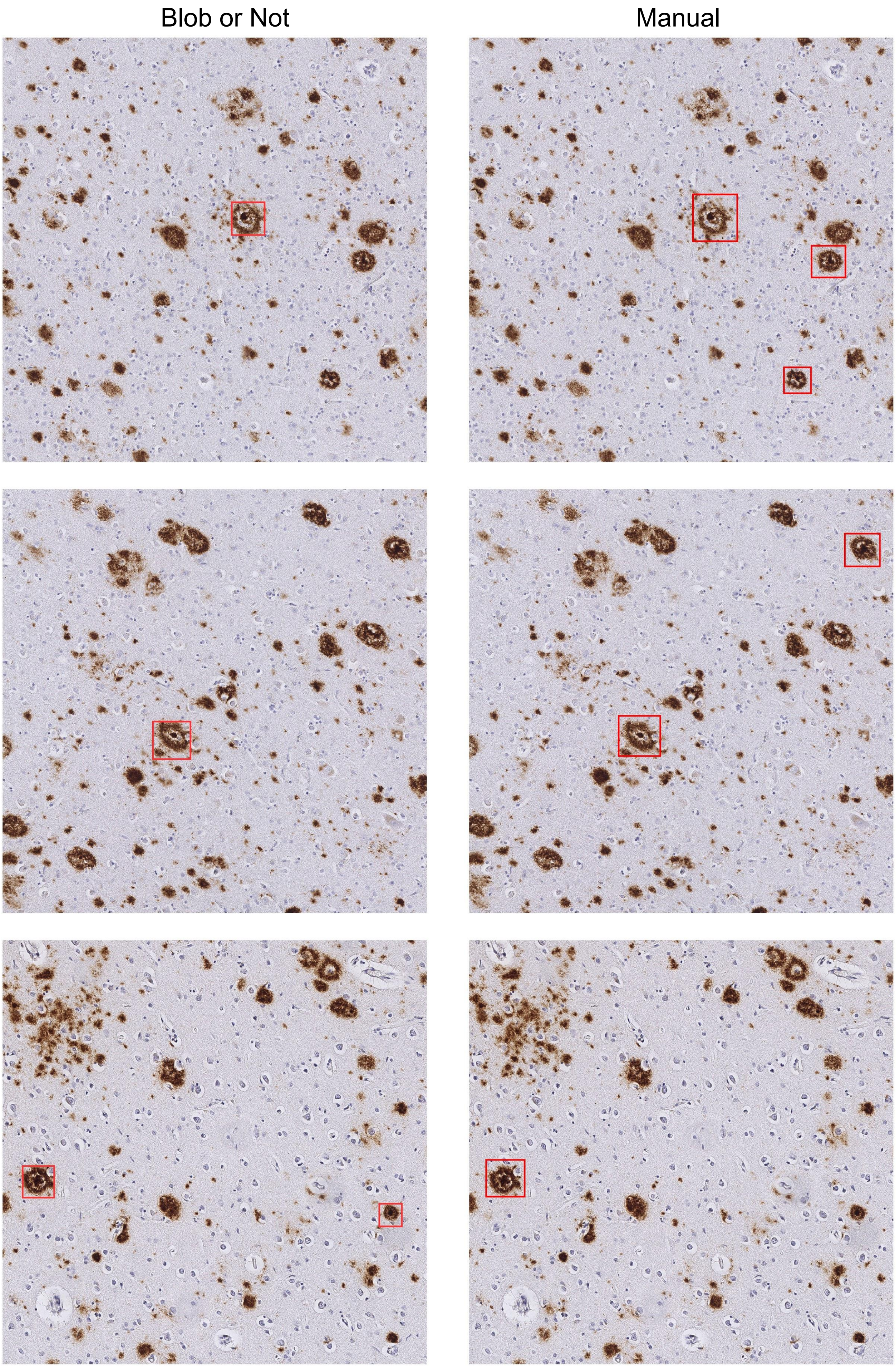

Supplementary Figure 11 Label comparison. Bounding boxes mark cored-plaque expert annotations

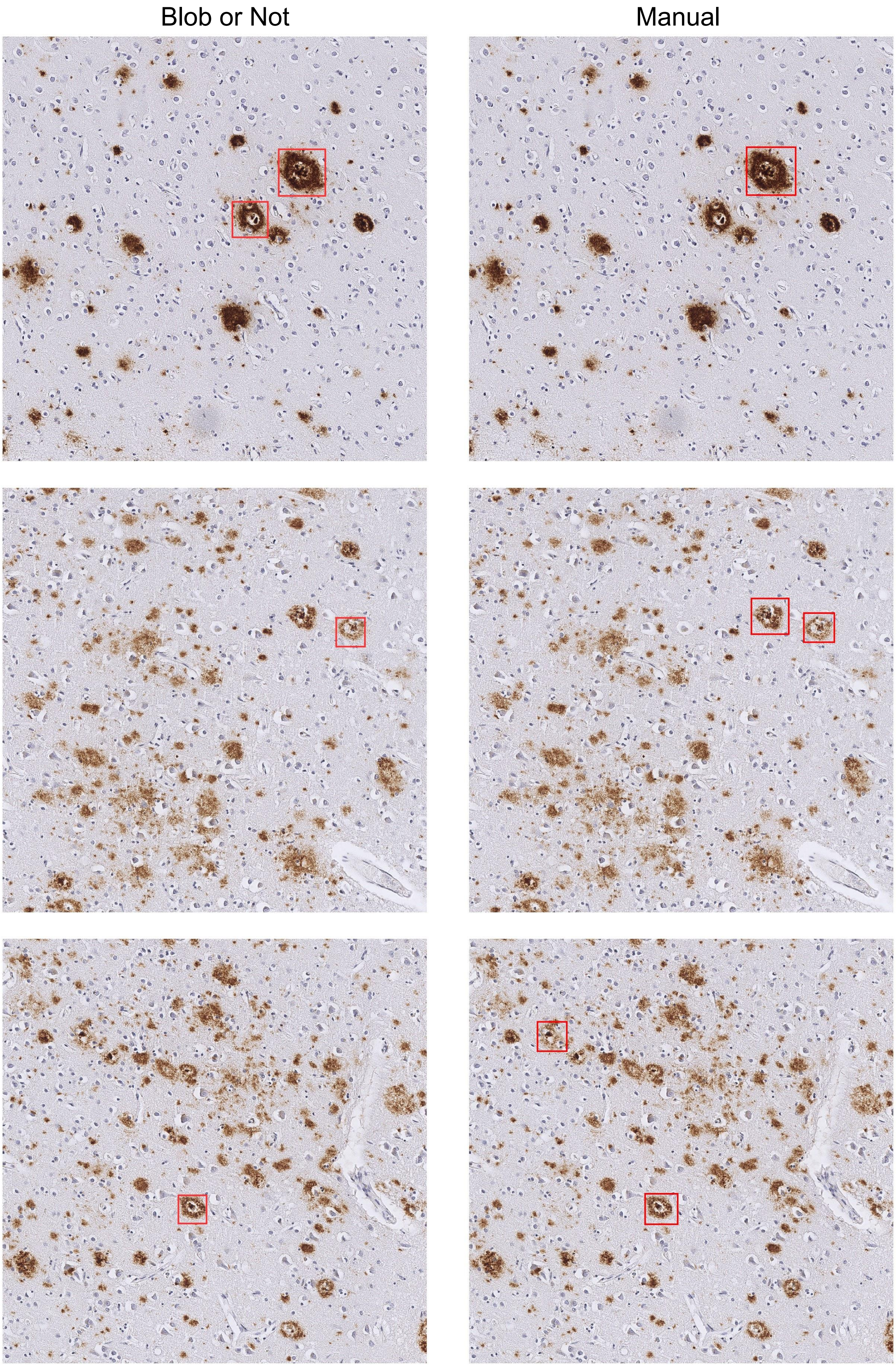

Supplementary Figure 11 continued

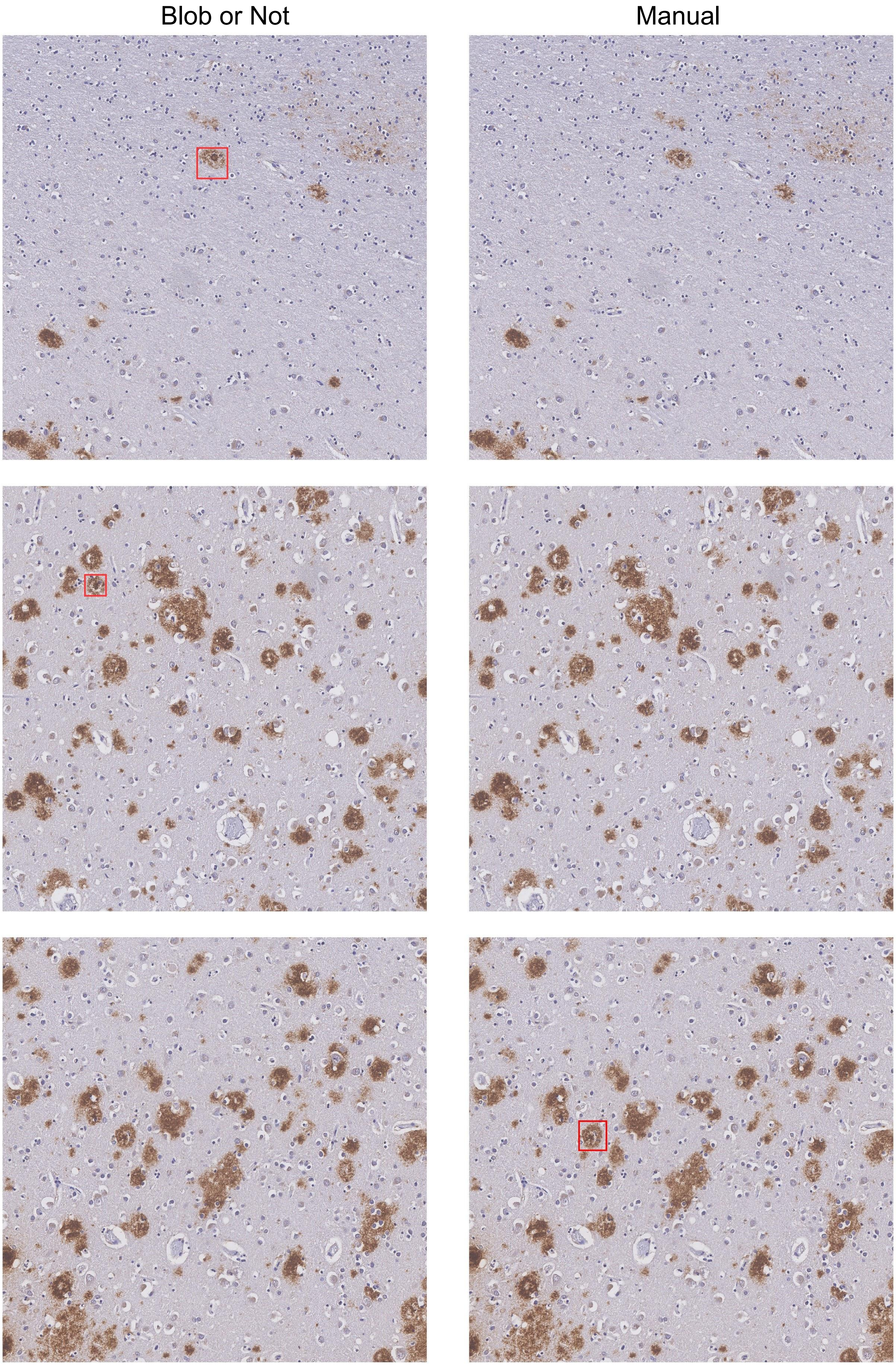

Supplementary Figure 11 continued

Supplementary Table 1 Demographic and neuropathology details of the initial dataset. Abbreviations: AD=Alzheimer's disease; CAA=cerebral amyloid angiopathy; CP=cored plaques; DP=diffuse plaques; hrs=hours; F=Female; M=Male; MTG=middle temporal gyrus; NP=neuritic plaque; PMI=post mortem interval; yrs=years; n/a=not available. For CERAD like scores 0=none, 1=sparse, 2=moderate, 3=frequent.
** As these were archival cases, sections were not available in all regions to determine the exact Thal Amyloid phase (Thal phase).*

Case #	Group	Age at Death (yrs)	Sex	PMI (hrs)	NIA Reagan	Thal Phase	Braak NFT stage	CERAD NP score	CAA type (1 or 2)	CERAD like Score CP MTG	CERAD like Score DP MTG	CERAD like Score CAA MTG
1	train	81	M	7.0	Not AD	at least Thal 3*	II	0	n/a	1	2	0
2	validation	74	M	19.0	Low	3	II	0	n/a	0	1	0
3	train	77	M	17.0	Not AD	1	II	1	n/a	0	1	0
4	train	79	F	4.6	Low	2	II	1	n/a	0	0	0
5	train	87	M	13.0	Low	2	II	1	n/a	0	0	0
6	train	77	F	2.1	Low	3	III	1	1	1	2	3
7	train	97	F	5.1	Low	1	III	1	1	1	1	2
8	train	83	M	5.0	Low	5	III	1	n/a	0	3	0
9	train	80	F	3.0	Intermediate	4	IV	2	n/a	1	2	0
10	hold out	81	F	5.0	High	at least Thal 2*	VI	2	n/a	2	2	0
11	train	82	M	19.0	High	4	VI	2	n/a	2	2	0
12	train	81	M	8.0	Intermediate	3	V	2	n/a	2	3	0
13	train	83	M	9.0	Intermediate	5	IV	2	1	0	2	3
14	train	88	F	35.0	High	5	V	2	2	2	2	1
15	train	88	F	9.0	High	3	V	2	n/a	0	0	0
16	train	91	F	6.8	Low	3	II	2	n/a	1	3	0
17	train	89	M	8.0	High	5	V	2	n/a	1	3	0
18	validation	88	M	19.0	High	4	VI	2	1	2	3	1
19	hold out	88	F	6.2	Intermediate	3	IV	2	n/a	1	2	0
20	train	62	M	7.6	High	5	VI	2	n/a	1	3	0
21	train	85	M	4.5	High	5	VI	2	2	1	3	2
22	train	89	F	20.0	High	2	V	3	n/a	0	0	0
23	hold out	76	F	3.3	High	4	V	3	n/a	1	2	0
24	hold out	78	F	7.0	High	5	V	3	n/a	2	3	0
25	hold out	91	F	5.0	High	5	VI	3	n/a	2	3	0
26	train	85	F	4.0	High	5	VI	3	n/a	2	2	0
27	train	77	M	8.0	High	5	V	3	n/a	2	3	0
28	hold out	81	M	4.0	High	5	VI	3	n/a	2	3	0
29	train	87	F	14.0	High	4	VI	3	1	2	3	1
30	train	62	F	4.0	High	5	V	3	n/a	3	2	0
31	train	83	M	2.0	High	5	IV	3	1	3	2	3
32	train	84	M	26.0	High	5	V	3	2	3	2	1
33	hold out	83	F	4.7	High	4	VI	3	2	1	3	2
34	train	87	F	31.0	High	5	VI	3	n/a	2	3	0
35	train	70	F	10.0	High	5	VI	3	n/a	1	3	0
36	validation	68	F	n/a	High	5	VI	3	1	2	3	1
37	validation	85	F	5.0	Intermediate	5	III	3	2	2	3	1
38	train	90	M	13.0	High	5	VI	3	n/a	1	3	0
39	hold out	78	F	6.0	High	5	VI	3	2	2	3	1
40	hold out	88	M	7.0	High	5	VI	3	n/a	1	2	0
41	hold out	95	M	42.0	High	5	IV	3	2	2	3	1
42	train	92	F	4.0	High	3	VI	3	1	1	2	1
43	train	n/a	n/a	n/a	n/a	n/a	n/a	n/a	n/a	2	2	0

Supplementary Table 2 Demographic and neuropathology details of additional hold out dataset. Abbreviations: AD=Alzheimer's disease; CAA=cerebral amyloid angiopathy; CP=cored plaques; DP=diffuse plaques; hrs=hours; F=Female; M=Male; MTG= middle temporal gyrus; NP=neuritic plaque; PMI=post mortem interval; yrs=years; n/a=not available. For CERAD like scores: 0=none, 1=sparse, 2=moderate, 3=frequent.

Case #	Group	Age at Death (yrs)	Sex	PMI (hrs)	NIA Reagan	Braak NFT stage	CERAD NP score	CERAD like Score CP MTG	CERAD like Score DP MTG	CERAD like Score CAA MTG
1	hold out	83	M	26	Low	II	0	0	0	0
2	hold out	77	M	6.75	Not AD	I	0	0	0	0
3	hold out	79	F	18	Low	I	0	1	1	0
4	hold out	72	M	4	Low	I	0	0	1	0
5	hold out	93	F	5	Low	II	1	1	1	0
6	hold out	96	F	6.95	Low	II	1	0	3	0
7	hold out	78	F	9	Low	III	1	2	2	3
8	hold out	87	M	5	Intermediate	III	1	0	3	3
9	hold out	92	F	4	Intermediate	IV	1	2	2	0
10	hold out	81	M	15	Intermediate	III	2	1	1	0
11	hold out	81	M	7	High	V	2	1	3	1
12	hold out	78	M	9	High	V	3	3	3	0
13	hold out	81	M	4	High	V	2	3	3	3
14	hold out	81	F	20	Intermediate	IV	3	2	2	0
15	hold out	90	F	76.5	High	VI	3	0	3	1
16	hold out	80	M	3	High	VI	3	2	3	0
17	hold out	89	M	7	High	V	3	2	3	1
18	hold out	89	M	n/a	High	VI	3	3	3	1
19	hold out	69	M	4	High	V	3	2	3	2
20	hold out	59	F	5	High	VI	3	2	3	0

Supplementary Table 3 Classification accuracy on validation set and hold-out test set. Using a threshold of 0.91, 0.1 and 0.85 for cored plaque, diffuse plaque, and CAA prediction respectively. Predictions with confidence above the threshold are considered to be positives.

	**Overall**	**Cored Plaque**	**Diffuse Plaque**	**CAA**
Validation set	0.973	0.978	0.949	0.993
Dataset size (*n*)	8,630	381	7,487	126
Hold-out test set	0.987	0.993	0.970	0.999
Dataset size (*n*)	10,873	98	10,480	7

Supplementary Table 4 Classification accuracy on validation set with different thresholding (see **Supplementary Table 3** for dataset sizes). Predictions with confidence above the threshold are considered to be positives.

Cored Plaque	Diffuse Plaque	CAA	Threshold
0.044	0.868	0.015	0.00
0.873	0.945	0.987	0.05
0.890	0.949	0.989	0.10
0.898	0.948	0.990	0.15
0.904	0.948	0.990	0.20
0.910	0.945	0.991	0.25
0.914	0.942	0.991	0.30
0.918	0.940	0.992	0.35
0.921	0.938	0.992	0.40
0.925	0.935	0.992	0.45
0.929	0.934	0.992	0.50
0.932	0.931	0.992	0.55
0.936	0.928	0.992	0.60
0.941	0.923	0.993	0.65
0.945	0.918	0.993	0.70
0.951	0.910	0.993	0.75
0.959	0.895	0.993	0.80
0.967	0.867	0.993	0.85
0.976	0.813	0.993	0.90
0.980	0.710	0.993	0.95

Supplementary Table 5 Classification accuracy on hold-out test set with different thresholding (see **Supplementary Table 3** for dataset sizes). Predictions with confidence above the threshold are considered to be positives.

**Cored Plaque**	**Diffuse Plaque**	**CAA**	**Threshold**
0.009	0.964	0.001	0.00
0.938	0.969	0.991	0.05
0.951	0.970	0.993	0.10
0.957	0.970	0.994	0.15
0.960	0.970	0.995	0.20
0.963	0.969	0.995	0.25
0.964	0.968	0.995	0.30
0.966	0.967	0.996	0.35
0.967	0.967	0.996	0.40
0.969	0.967	0.996	0.45
0.971	0.965	0.996	0.50
0.973	0.964	0.996	0.55
0.976	0.961	0.997	0.60
0.979	0.957	0.997	0.65
0.981	0.952	0.997	0.70
0.983	0.943	0.998	0.75
0.986	0.929	0.998	0.80
0.988	0.902	0.999	0.85
0.993	0.853	0.999	0.90
0.995	0.742	0.999	0.95

Supplementary Table 6 Spearman rank-order correlation coefficient between model-based whole-slide scores and CERAD categories. Sample size n = 62 for entire dataset, n = 30 for blinded hold-out set.

	Cored plaque	Diffuse plaque	CAA
Entire dataset	0.82	0.74	0.70
Blinded hold-out set	0.81	0.76	0.72
