# Supplementary material for: Interpretable classification of Alzheimer’s disease pathologies with a convolutional neural network pipeline
Source: Nat Commun. 2019 May 15;10:2173. doi: 10.1038/s41467-019-10212-1 (PMC6520374; doi:10.1038/s41467-019-10212-1)
Supplement: Supplementary file 3 — Reporting Summary [file 41467_2019_10212_MOESM3_ESM.pdf]

nature research

Corresponding author(s): Michael J. Keiser, PhD and Brittany N. Dugger, PhD

Last updated by author(s): Mar 28, 2019

Reporting Summary

Nature Research wishes to improve the reproducibility of the work that we publish. This form provides structure for consistency and transparency in reporting. For further information on Nature Research policies, see Authors & Referees and the Editorial Policy Checklist.

Statistics

For all statistical analyses, confirm that the following items are present in the figure legend, table legend, main text, or Methods section.

n/a	Confirmed	
☐	☒	The exact sample size (*n*) for each experimental group/condition, given as a discrete number and unit of measurement
☐	☒	A statement on whether measurements were taken from distinct samples or whether the same sample was measured repeatedly
☐	☒	The statistical test(s) used AND whether they are one- or two-sided<br>*Only common tests should be described solely by name; describe more complex techniques in the Methods section.*
☒	☐	A description of all covariates tested
☐	☒	A description of any assumptions or corrections, such as tests of normality and adjustment for multiple comparisons
☐	☒	A full description of the statistical parameters including central tendency (e.g. means) or other basic estimates (e.g. regression coefficient) AND variation (e.g. standard deviation) or associated estimates of uncertainty (e.g. confidence intervals)
☐	☒	For null hypothesis testing, the test statistic (e.g. *F*, *t*, *r*) with confidence intervals, effect sizes, degrees of freedom and *P* value noted<br>*Give P values as exact values whenever suitable.*
☒	☐	For Bayesian analysis, information on the choice of priors and Markov chain Monte Carlo settings
☒	☐	For hierarchical and complex designs, identification of the appropriate level for tests and full reporting of outcomes
☒	☐	Estimates of effect sizes (e.g. Cohen's *d*, Pearson's *r*), indicating how they were calculated

Our web collection on statistics for biologists contains articles on many of the points above.

Software and code

Policy information about availability of computer code

Data collection	All whole slide images were digitized using an Aperio AT2 and Aperio ImageScope software. Custom software written in Python was developed using the open-source packages PyVips and OpenCV. Custom software for deep learning models were trained in the open-source library PyTorch.
Data analysis	Code is available at GitHub (https://github.com/keiserlab/plaquebox-paper/).

For manuscripts utilizing custom algorithms or software that are central to the research but not yet described in published literature, software must be made available to editors/reviewers. We strongly encourage code deposition in a community repository (e.g. GitHub). See the Nature Research guidelines for submitting code & software for further information.

Data

Policy information about availability of data

All manuscripts must include a data availability statement. This statement should provide the following information, where applicable:

- Accession codes, unique identifiers, or web links for publicly available datasets
- A list of figures that have associated raw data
- A description of any restrictions on data availability

We have made the CNN model demo code (https://github.com/keiserlab/plaquebox-paper/) and the full raw WSI dataset and the annotated plaque-level dataset (https://doi.org/10.5281/zenodo.1470797) openly available. A landing page is available at https://www.keiserlab.org/resources.

Field-specific reporting

Please select the one below that is the best fit for your research. If you are not sure, read the appropriate sections before making your selection.

☒ Life sciences ☐ Behavioural & social sciences ☐ Ecological, evolutionary & environmental sciences

For a reference copy of the document with all sections, see nature.com/documents/nr-reporting-summary-flat.pdf

Life sciences study design

All studies must disclose on these points even when the disclosure is negative.

Sample size	77,000 unique 256x256-pixel image tiles were extracted from 43 whole slide images, each representing a unique decedent case. A further 20 whole slide images, representing further unique cases, were analyzed for slide-level CERAD-like scores in Figure 8.
Data exclusions	No data were excluded from the study.
Replication	All code and data used in this study are made openly available for reproducibility.
Randomization	All code was run with random seeds for reproducibility. Whole slide images for training and validation were randomly split on a per-case basis.
Blinding	All slides from decedent cases were anonymized using a unique identifier code. The semi-manual CERAD-like scores of 20 held-out whole slide images were blinded in Figure 8b.

Reporting for specific materials, systems and methods

We require information from authors about some types of materials, experimental systems and methods used in many studies. Here, indicate whether each material, system or method listed is relevant to your study. If you are not sure if a list item applies to your research, read the appropriate section before selecting a response.

Materials & experimental systems

n/a	Involved in the study	
☐	☒	Antibodies
☒	☐	Eukaryotic cell lines
☒	☐	Palaeontology
☒	☐	Animals and other organisms
☒	☐	Human research participants
☐	☒	Clinical data

Methods

n/a	Involved in the study	
☒	☐	ChIP-seq
☒	☐	Flow cytometry
☒	☐	MRI-based neuroimaging

Antibodies

Antibodies used	Amyloid-β antibody (4G8, recognizing residues 17-24, dilution 1:1600, BioLegend (formally covance), catalog number SIG-39200)
Validation	4G8 antibody staining was conducted at the University of California Department of Pathology and Laboratory Medicine Research Histology Laboratory. The laboratory is both CLIA and CAP accredited. Proper controls were utilized for all staining. Antibody product web page: https://www.biolegend.com/en-us/products/purified-anti-beta-amyloid--17-24-antibody-11233

Clinical data

Policy information about clinical studies

All manuscripts should comply with the ICMJE guidelines for publication of clinical research and a completed CONSORT checklist must be included with all submissions.

Clinical trial registration	n/a
Study protocol	Study did not involve human research participants--the only clinical data used was the presence of dementia. To determine dementia, all subjects received multidisciplinary diagnostic evaluations at enrollment and approximately annual intervals thereafter until death, loss to follow-up, or inability to return for reassessment due to disability. Details of this program have been previously published (Hinton et al. 2010 Alzheimer Dis Assoc Disord 24, 234–241).
Data collection	The only clinical data used was the presence of dementia. See Study protocol field for details. Data was collected using standard forms from the National Alzheimer's Coordinating Center (https://www.alz.washington.edu/WEB/forms_uds.html).
Outcomes	n/a
